# The Strange Lifestyle of Multipartite Viruses

**DOI:** 10.1371/journal.ppat.1005819

**Published:** 2016-11-03

**Authors:** Anne Sicard, Yannis Michalakis, Serafín Gutiérrez, Stéphane Blanc

**Affiliations:** 1 INRA, UMR BGPI, Montpellier, France; 2 CNRS, UMR MIVEGEC 5290, Montpellier, France; 3 CIRAD, UMR CMAEE, Montpellier, France; University of Alberta, CANADA

## Abstract

Multipartite viruses have one of the most puzzling genetic organizations found in living organisms. These viruses have several genome segments, each containing only a part of the genetic information, and each individually encapsidated into a separate virus particle. While countless studies on molecular and cellular mechanisms of the infection cycle of multipartite viruses are available, just as for other virus types, very seldom is their lifestyle questioned at the viral system level. Moreover, the rare available “system” studies are purely theoretical, and their predictions on the putative benefit/cost balance of this peculiar genetic organization have not received experimental support. In light of ongoing progresses in general virology, we here challenge the current hypotheses explaining the evolutionary success of multipartite viruses and emphasize their shortcomings. We also discuss alternative ideas and research avenues to be explored in the future in order to solve the long-standing mystery of how viral systems composed of interdependent but physically separated information units can actually be functional.

## Introduction

The architecture, organization, and packaging of viral genetic information can be divided into three categories: monopartite, segmented, and multipartite viruses. Monopartite viruses have a single nucleic acid molecule protected in a shell made of proteins (and sometimes also lipids) forming the virus particle. The genome of segmented viruses is divided into two or more nucleic acid segments that are all encapsidated together in a single virus particle. Multipartite viruses (the terms multicomponent viruses and coviruses are also used in the literature) have their genome divided into two or more nucleic acid segments, just as the segmented type, but these segments are each packaged into separate virus particles. This latter peculiar organization is the only one resulting in viral transmissible entities that do not contain the entire genetic information, and in which the co-transmission of several virus particles to a new cell or host appears mandatory to maintain the integrity of the viral genome. The biology of multipartite viruses challenges some basic concepts of virology and evolution, and, at this point, it remains hard (if possible at all) to conceive how they have evolved and how they can actually be functional.

The report of so-called multicomponent viruses marked an important step in the history of the discovery of viruses. When analytical centrifugation techniques emerged [1], it was rapidly noted that some viral-like diseases were associated to two or more protein and nucleic acid components of different density. The first confirmed cases were *Tobacco rattle virus* [2] and *Cowpea mosaic virus* [3], but at that time their multicomponent nature could not be understood. Together with earlier dose-related infectivity studies ([4] and references within), further development of biochemistry, electron microscopy, molecular biology, and sequencing definitely evidenced that many viruses are composed of two or more physically separated particles, each containing a complementary portion of the genetic information [5, 6].

While monopartite and segmented viruses infect all possible living organisms, multipartite viruses appear mostly restricted to plants and fungi. Thus far in animals, the only viral species demonstrated to be multipartite as defined here are the ssDNA bidensoviruses in silkworm [7] and a very recently reported ss(+)RNA virus in mosquitoes [8]. Note that the polydnaviruses of insects are not considered here as multipartite viruses. Their genome is integrated into that of their parasitic wasp host, where it is transferred vertically together with the wasp genome. These viruses are never transmitted horizontally as an episomal replication-competent entity [9]. That 30%–40% of plant virus genera and families are multipartite [10] is a long-standing mystery. It had early been speculated that the multipartite architecture of the genome could be related to its RNA nature and had been proposed that multipartite viruses are so frequent in plants because most plant viruses are RNA viruses [11]. However, it is now clear that DNA multipartite viruses are also frequent among plant viruses. In fact, multipartite viruses can be (+)ssRNA, (-)ssRNA, dsRNA, and ssDNA viruses, their genome size is highly variable, they can form icosahedral, rod-like, or filamentous virus particles, and none of these features demarcates them from monopartite viruses (Table 1).

**Table 1:** Summary of the families and genera of plant viruses with distinct virion structure, genome nature, and organization.

Genome^1^		Family	Genus	Particle^2^	Segments^3^	Mono.^4^	Seg.^4^	Multi.^4^
DNA	ssDNA	*Geminiviridae*	*Begomovirus* ^5^	*twinned icosahedra*	*1 to 2*	*✔*		*✔*
			*all other genera*		*1*	*✔*		
		*Nanoviridae*	*Babuvirus*	*icosahedra*	*6*			*✔*
			*Nanovirus*		*8*			*✔*
	dsDNA	*Caulimoviridae*	*Caulimovirus & all other genera*	*icosahedra*	*1*	*✔*		
			*Badnavirus & Tungrovirus*	*bacilliform*	*1*	*✔*		
RNA	ssRNA (-)	*Bunyaviridae*	*Tospovirus*	*enveloped spherical*	*3*		*✔*	
		*unassigned*	*Emaravirus*	*enveloped spherical*	*4*		*✔*	
		*Ophioviridae*	*Ophiovirus*	*flexuous nucleocapsid*	*3 to 4*			*✔*
		*unassigned*	*Tenuivirus*	*flexuous nucleocapsid*	*4 to 5*			*✔*
		*Rhabdoviridae*	*Cytorhabdovirus & Nucleorhabdovirus*	*enveloped*, *bullet-shaped*	*1*	*✔*		
		*unassigned*	*Varicosavirus*	*rod-shaped*	*2*			*✔*
	ssRNA (+)	*Closteroviridae* ^5^	*Closterovirus*	*filamentous*	*1*	*✔*		
			*Ampelovirus*		*1*	*✔*		
			*Crinivirus*		*2*			*✔*
		*Potyviridae* ^5^	*Potyvirus*, *Brambyvirus*, *Poacevirus*	*filamentous*	*1*	*✔*		
			*Bymovirus*		*2*			*✔*
			*Tritimovirus*, *Rymovirus*, *Ipomovirus & Macluravirus*		*1 or 2*	*✔*		*✔*
		*Alphaflexiviridae*	*all genera*	*filamentous*	*1*	*✔*		
		*Betaflexiviridae*	*all genera*	*filamentous*	*1*	*✔*		
		*Tymoviridae*	*all genera*	*icosahedra*	*1*	*✔*		
		*Benyviridae*	*Benyvirus*	*rod-shaped*	*4 to 5*			*✔*
		*Virgaviridae* ^5^	*Furovirus*	*rod-shaped*	*2*			*✔*
			*Hordeivirus*		*3 or 4*			*✔*
			*Pecluvirus*		*2*			*✔*
			*Pomovirus*		*3*			*✔*
			*Tobamovirus*		*1*	*✔*		
			*Tobravirus*		*2*			*✔*
		*Secoviridae* ^5^	*Sequivirus*	*icosahedra*	*1*	*✔*		
			*Waikavirus*		*1*	*✔*		
			*Comovirinae*, *Comovirus*, *Fabavirus*, *Nepovirus*		*2*			*✔*
			*Cheravirus*		*2*			*✔*
			*Sadwavirus*		*2*			*✔*
			*Torradovirus*		*2*			*✔*
		*Bromoviridae*	*Alfamovirus*, *Oleavirus*	*Bacilliform*	*3*			*✔*
			*Anulavirus*, *Bromovirus*, *Cucumovirus*, *Ilarvirus*	*icosahedra*	*3*			*✔*
		*Luteoviridae*	*all genera*	*icosahedra*	*1*	*✔*		
		*unassigned*	*Polemovirus*	*icosahedra*	*1*	*✔*		
		*unassigned*	*Sobemovirus*	*icosahedra*	*1*	*✔*		
		*Tombusviridae* ^5^	*Dianthovirus*	*icosahedra*	*2*			*✔*
			*all other genera*		*1*	*✔*		
		*unassigned*	*Idaeovirus*	*icosahedra*	*3*			*✔*
		*unassigned*	*Cilevirus*	*bacilliform*	*2*			*✔*
		*unassigned*	*Ourmiavirus*	*bacilliform*	*3*			*✔*
		*unassigned*	*Umbravirus*	*no capsid*, *satelite virus*	*1*	*✔*		
	dsRNA	*Endornaviridae*	*Endornavirus*	*none reported*	*1*	*✔*		
		*Partitiviridae*	*all genera*	*Icosahedra*	*2*			*✔*
		*Reoviridae*	*Fijivirus & Orizavirus*	*double layer icosahedra*	*10*		*✔*	
			*Phytoreovirus*		*12*		*✔*	
		*Metaviridae*	*Metavirus*	*Spherical irregular*	*1*	*✔*		
		*Pseudoviridae*	*Pseudovirus*	*spheroïd*, *ovoïd*	*1*	*✔*		

^1^ Nature of the nucleic acid composing the genome.

^2^ Type and shape of the virus particle.

^3^ Number of genome segments comprising the viral genome.

^4^ Mono., Seg., and Multi. correspond to monopartite, segmented, and multipartite viral species, as marked in corresponding boxes.

^5^ Note that the families *Virgaviridae*, *Potyviridae*, *Tombusviridae*, *Secoviridae*, and *Closteroviridae*, as well as the genus *Begomovirus*, are composed of both monopartite and multipartite species.

This table is adapted from the website "ViralZone" (http://viralzone.expasy.org/) and from *King*, *A*. *M*. *(2011)*. *Virus taxonomy*: *classification and nomenclature of viruses*: *Ninth Report of the International Committee on Taxonomy of Viruses (Vol*. *9)*. *Elsevier*, *p*. *18–19*.

Soon after its discovery, virologists and evolutionary biologists began to investigate the possible benefits and costs of such genome organization. The proposed, and sometimes disputed, benefits are mostly related to the smaller size of the genome segments, as opposed to a larger single molecule. Smaller segments may induce better tolerance to high mutation rates [11], faster replication [12], facilitated genetic exchange between an increased number of small information modules, each represented by a different segment [13], or a higher stability of viral particles [14]. It is worth noting that all these putative benefits (except perhaps the last one) apply equally to segmented viruses; they can thus potentially explain the benefits of having a divided genome but not the very nature of a multipartite life: distinct packaging of the different genome pieces.

The counterpart cost opposed to these benefits is the reduced chance to infect new cells (and new hosts) with all components required for functionality/integrity of the viral genome [11–13],[15]. This probability decreases with the number of genome segments and, thus, of virus particles needed to recapitulate the entire genome. Furthermore, for a given number of genome segments, the cost is higher when their relative frequencies differ, i.e., when some segments are rare. In contrast to the arguable benefits, this cost is so intuitive that it is never disputed and equally assumed in all studies modeling the evolution of multipartite viruses.

A main concern in this field of research is that most of the above cited studies are theoretical and that experimental support for the proposed benefits and costs is rare, if available at all. When considering the common wisdom on viruses, it is clear that a conceptual frame derived from the understanding of canonical monopartite viruses largely dominates. Possibly, this frame biases the conception of theoretical models intended to explain the evolution of multipartite viral systems and hampers the design of experiments that would relevantly address biological processes specifically adapted to the way of life of multipartite viruses.

Below, we briefly review the successive steps of the life cycle of viruses, trying to extract and highlight empirical or theoretical data specifically relevant for the biology of multipartite viruses. We thereby identify major gaps and future research lines that would allow a better comprehension of these intriguing biological systems.

### 1. Replication

Multipartite viruses, just as monopartite and segmented viruses, are replicated through a diversity of mechanisms depending partly on their genome nature. Whatever the molecular details or the cellular location of viral replication, two specific benefits have been proposed and modeled to explain the evolution of genome segmentation, thus of both segmented and multipartite viruses. The first putative replication-related benefit is that splitting a genome into several smaller segments should result in faster replication [12],[16,17]. This is based on the trivial fact that, when the speed of the replicase is constant and when this replicase is not a limiting factor, the time required for the duplication of a genome of 10 kb is twice longer than that for two segments of 5 kb each. Experimental studies looking at the replication kinetics as a general function of genome length seem to corroborate this hypothesis [16,17],[18]. However, the only study directly comparing a monopartite genome to its bipartite derivative obtained in infected cell cultures could not confirm the expected faster replication of the bipartite variant [14]. This contrasting report calls for more studies comparing near-isogenic monopartite and multipartite viruses, in order to empirically confirm that faster replication can indeed benefit segmented genomes. In addition, while faster replication can be a competitive advantage in simple theoretical models and experimental designs, the existence of trade-offs (as that between virulence and transmission) in more realistic host and ecological contexts might mitigate its beneficial effects (see [19]).

The second replication-related benefit proposed for segmented genomes is that smaller segments are smaller targets for mutations [11,13]. In this hypothesis, several small segments could each generate non-mutated offspring when a full-genome-length molecule could not. Initially invoked for RNA viruses replicated by error-prone RNA-dependent RNA-polymerases [20], this argument could now be extended to ssDNA viruses of plants (Gemini- and nanoviruses) and animals (circo-, denso-, and parvoviruses), in which mutation, substitution, and evolution rates comparable to that of RNA viruses have been repeatedly documented (for examples see: [21–23] and references therein). Different studies comparing the mutation rate in distinct families of RNA viruses have suggested a possible negative correlation between mutation rate and genome length [24,25], indicating that smaller genomes or segments can tolerate higher mutation frequencies. In contrast, a comprehensive recent report has compiled a large dataset of 118 substitution rates, from 91 genes of 28 viral species [26]. By testing the relationship between substitution rates, several viral genome properties, and ecological factors, the authors concluded that the nature of the target cells is the only significant predictor of viral substitution rates, whereas genome length and genome segmentation are not.

Altogether, it seems reasonable to conclude that the currently available data do not corroborate the hypotheses explaining the evolution of genome segmentation by a faster replication and/or an easier mutational escape of smaller genome segments. In addition, whatever the future outcome of this debate, the arguments apply to both segmented and multipartite viruses and, thus, cannot explain the separate encapsidation of segments in multipartite viruses.

As opposed to the above arguable benefits, one replication-associated constraint for segmented genomes is the need to bear similar origins of replication and regulatory elements on all segments in order to be efficiently recognized and processed by the replication complex. The existence of conserved origins of replication among genome segments has been demonstrated for a number of segmented and multipartite viruses, as, for example, bromoviruses [27,28], begomoviruses [29], and nanoviruses [30]. A deletion/mutation in this region drastically affects the replication efficiency of the corresponding segments, and, thus, in order to coordinate the various functions distributed across distinct segments, its concerted evolution is mandatory [31]. While the conservation of these regulatory sequences may appear as a burden for segmented and multipartite viral genomes, it might also promote genetic exchanges, as discussed in the next section.

### 2. Genetic exchanges

The genetic exchanges between viral genomes can depend directly on the replication mechanisms. However, they can also be replication-independent, through genome break/repair processes and through reassortments in segmented and multipartite viruses.

A reassortment can be defined as an exchange of homologous segments between two related virus isolates or species; thus, a form of genetic exchange not involving intramolecular crossovers. Reassortment had been suggested as a substitute for intramolecular recombination in segmented RNA genomes at a time when RNA crossovers were believed to be rare, if not impossible [11,32]. Thus, for RNA viruses, reassortment was perceived as the major means to promote genetic exchange and to both purge deleterious mutations and create new advantageous combinations [13].

Numerous studies describe the existence of reassortants from the analysis of sequence datasets and postulate that they play an important role in the evolutionary history of the corresponding viruses. Specific reassortants were shown or suggested to have a higher fitness in segmented and multipartite viruses of animals and plants, such as, for example, *Influenza virus*, *Bluetongue virus*, *Tomato spotted wilt virus*, *Cucumber mosaic virus*, and several nanoviruses [31,33–39]. Some of these studies show, however, that the potential advantage of reassortment can be limited to specific portions of the viral genome. For instance, in *Influenza virus* or several plant nanoviruses, reassortments seem to be favored for one or two segments, whereas other possible combinations more rarely emerge [40],[31,37,38].

In 1986, Bujarski and colleagues [41] demonstrated that *Brome mosaic virus* (BMV), a multipartite (+)ssRNA plant virus, could also recombine by intramolecular RNA crossovers. More recently, it became evident that most (+)ssRNA viruses have recombination rates as high as DNA viruses [38,42–44], and that intrasegment recombinants are as frequent as inter-segment reassortants in multipartite viruses [37, 38, 45, 46]. From this wealth of new data, it is now evident that both recombination and reassortment promote frequent genetic exchanges in multipartite viruses. How this is affecting the hypothesis that genome segmentation has evolved to allow sex through reassortment is unclear, but the demonstrated ease of intramolecular recombination may mitigate some of its authority.

The mechanisms of intramolecular recombination have *a priori* no reason to differ between monopartite, segmented, and multipartite viruses, and are thus not detailed further. In contrast, as mentioned in the section above, the role of the conserved replication origin in distinct segments of the same viral genome deserves mention in this section because it promotes homology-driven intersegment recombination. This has been experimentally forced under high selection pressure for the multipartite *Brome mosaic virus* [41] and also observed in natural populations for the multipartite genus *Nanoviridae* [37,38]. Because these replication origins are sometimes conserved in different species of the same family, homologous-recombination events can paradoxically occur between heterologous segments originating from distinct species, as previously observed in field-collected bromo- and nanovirus samples [28,30]. One could argue that such recombination between segments encoding distinct functions is nonsense. However, a sound “raison d’être” could be the facilitation of the exchange of replication origins between segments of related genomes [31,47]. This might help spreading reassorted segments from distinct strains or species by rapidly matching their replication origin to the viral system into which they become incorporated.

### 3. Gene expression

Viruses have been extremely inventive in order to encode all required genes and functions in very small genomes and to regulate their coordinated expression. In particular, viruses have to deal with the host cell machinery, which generally limits the translation of mRNAs to only one ORF. Over ten distinct strategies have been described for gene expression of monopartite viruses, and most are also used by segmented and multipartite viruses (reviewed in [10]). For example, viral genomes or segments can encode for a single or several genes; these genes can be expressed from a single mRNA or from subgenomic (or subsegment) mRNAs, as a single protein that can sometimes be subsequently cleaved in several products with specific functions. In other cases, the genomes or segments can have internal ribosome entry sites (IRES) allowing several ORF to be translated, leaky scanning sequences allowing polycistronic RNA to produce more than one protein, or strategies to promote reinitiation of translation of several ORFs encoded on a single mRNA.

In front of this complexity, the viral genome segmentation could provide an extreme simplification in which each segment would encode for a single gene. A long-foreseen advantage of segmented and multipartite viral systems would be that each segment could possess its own specific regulatory sequence [48],[49]. Surprisingly enough, only two virus groups have evolved this ultimate simplification: the multipartite virus families *Nanoviridae* and *Partitiviridae*. In all other segmented or multipartite virus species, the one-segment/one-gene strategy is either not found or combined with other segments encoding multiple genes. In conclusion, the hypothesis that the genome segmentation could be a simple way to match the gene expression and RNA translation machineries of the host cell does not seem totally satisfactory, because in most cases the control of gene expression in these viruses is observed to be as complex as in monopartite viruses.

**Fig 1:**
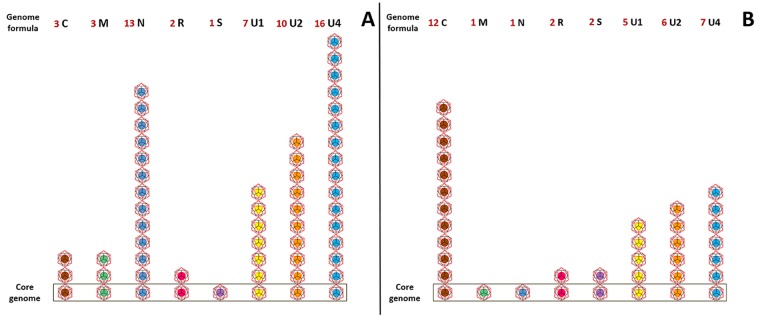
Schematic representation of the genome formula of *Faba bean necrotic stunt virus* (FBNSV) in two different host plant species. The genome formulae presented are in *Vicia faba* (A) and *Medicago truncatula* (B). The relative frequencies of the eight FBNSV segments have been calculated in within-host viral populations. The rounded median copy number of each segment is represented relative to the less abundant segment, here arbitrarily set to one. The core genome corresponds to the classical conception of a viral genome (rectangle). Adapted from reference [50].

One additional way to regulate gene expression that is readily possible in segmented and multipartite, but not so in monopartite, viruses is the differential regulation of gene (or segment) copy numbers [50–52]. Despite an important literature on the significant impact of gene copy number (GCN) variations on gene expression in all cellular organisms [51],[53],[54], this idea has thus far hardly made its way into virology [50,52],[55], perhaps due to the fact that copy number variations appear poorly amenable in size-constrained viral genomes. However, this view can change if the level at which gene copy number varies is not the individual genome but a population of segments. Until recently, the relative frequency of the different genome segments and, thus, their relative copy number in a within-host viral population had not been explicitly estimated, in either segmented or multipartite viruses. We directly addressed this question in the nanovirus *Faba bean necrotic stunt virus* (FBNSV) [50] and found that each ssDNA segment (so in this case each gene) accumulates reproducibly with a specific relative copy number in a given environment. We proposed that these copy numbers, each associated to a specific segment, define the “genome formula” (Fig 1A), which proved to be specific to the host plant species (Fig 1B). Because viral populations closer to the steady state or setpoint genome formula accumulated to higher levels, we hypothesized that the differential regulation of GCN might be adaptive and could stand as an unforeseen benefit for multipartite viruses. Several earlier hints indicating that multipartite viruses other than nanoviruses could also regulate GCN are discussed in [50], and a direct demonstration has been published recently for the tripartite (+)ssRNA *Alfalfa mosaic virus* [56]. While multipartite viruses can potentially control GCN at all steps of their life cycle (replication within cell, transmission to next cell and to next host), segmented viruses appear constrained at the transmission steps by the fact that in most cases, a single copy of each segment is encapsidated in each virus particle [57–62]. Segmented viruses could, however, control GCN at the intracellular level and at the within-host population level by specifically modifying the efficiency of packaging of one gene segment, but this latter possibility thus far relies on very rare publications [63].

The discovery of the genome formula might represent a significant step forward in the understanding of the specific biology of multipartite viruses for several reasons: (i) it represents an unprecedented putative advantage for the regulation of gene expression in segmented viral genomes; (ii) this advantage applies best to multipartite viruses because GCN could easily be regulated at all infection steps; (iii) as further discussed later, this advantage is the only one described thus far that imposes a constraint on the relative frequency of the segments, which can explain why these viral systems have not evolved to the situation of the minimum cost at which all segments would ideally accumulate at equal frequency.

### 4. Encapsidation

This step of the virus life cycle is extremely important in this review because it markedly distinguishes the segmented from the multipartite viruses. These are seemingly opposite strategies: in the former, the virus packages its entire genetic information in a single particle, thus enforcing that it travels together within hosts and from host to host; in the latter, apparently not.

Segmented viruses have a specific constraint at encapsidation, which is the sorting of distinct segments to ensure that at least one of each is present in every single particle. The molecular means by which this process is accomplished are partly understood. The best-documented cases are those of the *Influenza virus* and of phage Phi6. The segments bear different and complementary packaging signal sequences, which induce specific secondary RNA folding and a timely concerted interaction with the structural protein, ensuring the sorting of one copy of each segment per virion. Although such a precisely regulated process is highly efficient in phage Phi6 [64], it appears more variable in the case of *Influenza virus*, in which some studies reported virions with a complete set of segments [61,62] and others have evidenced virions with missing segments and/or duplicated ones [63,65]. A remarkable contrasting case is that of the *Infectious bursal disease virus*, with a genome made of two RNA segments but enough space in the virion to encapsidate up to four. This extra space allows co-packaging of multiple copies of the two segments with no need for specific sorting, apparently randomly ensuring a sufficiently high proportion of the generated virus particles with at least one copy of each segment [66]. A similar non-selective packaging of the three segments of *Rift valley fever virus* has been recently demonstrated [67].

*A priori* multipartite viruses do not have to sort segments at encapsidation; they could encapsidate using similar mechanisms as monopartite viruses, the frequency of encapsidated segments directly depending on the frequency of these segments within producing cells. As for monopartite viruses, the specific packaging of viral segments of multipartite genomes relies on the presence of assembly signal sequences [68–70]. A noticeable difference with monopartite viruses, however, is that the same coat protein(s) has to accommodate the packaging of segments of different sizes, sequences, and secondary/tertiary structures. Multipartite virus particles can be either spherical (*Partitiviridae*, *Nanoviridae*, *Begomovirus*, *Secoviridae*, *Idaeovirus*, etc.), bacilliform (*Ourmiavirus*, *Alfamovirus*, etc.), rod-shaped (*Virgaviridae*, *Varicosavirus*, *Benyvirus*, etc.) or filamentous (*Closteroviridae*, *Potyviridae*)(Table 1). While rod-shaped, bacilliform, and filamentous viruses can easily accommodate segments of different sizes by accordingly adjusting the length of the virus particles, physical constraints exist for viruses with an icosahedral structure. A nice illustration of this constraint is found in in vitro studies of particles made with the coat protein of *Cowpea chlorotic mottle virus* (CCMV) with a symmetry T = 3 [71]. The particles can encapsidate segments from 100 to 12,000 base-long RNAs, but they preferentially package one or more RNA segments with a total size around 3.2 kb, consistently resulting in an optimal protein/RNA ratio of 6/1 that corresponds to the natural situation for this virus.

In some icosahedral viral species, all segments have a comparable size, indicating that this size may be optimal for efficient packaging and particle stability. This is particularly striking for ssDNA multipartite nanoviruses, in which the eight genome segments vary between 920 and 1,022 nt in all described species. The ssDNA bipartite geminiviruses of plants and bidensoviruses of insects also have two genomic segments of similar size of around 2.7 kb for the first and 6 kb for the second. In other cases, however, the size of segments encapsidated in icosahedral particles can widely vary. In many species of the family *Bromoviridae*, the RNAs 1, 2, 3, and 4 are approximately of 3, 3, 2, and 1 kb, respectively. This is somehow intriguing because it could theoretically allow the encapsidation of two or more short segments in a single particle. A case study illustrating this is that of *Brome mosaic virus* (BMV), in which some particles contain either one copy of RNA-1 or one copy of RNA-2 (around 3 kb each), whereas others contain one copy of RNA-3 and one of RNA-4, together also summing up to approximately 3 kb [72,73]. It was recently shown that the situation in BMV is even more complex, because some smaller virus particles have been found and might also accommodate a single copy of either RNA-3 or RNA-4 [74].

Once encapsidated, the different interactions occurring between the distinct BMV genome segments and the capsid, resulting from distinct 3D structure of the packaged RNA, can engender different virus particle stabilities [75]. This difference could actually constitute an advantage of multi-encapsidation, because it may regulate a differential timing of RNA release and, thus, the kinetics of gene expression [74,75]. Likewise, in rod- or filamentous-shaped particles, the possible time shift associated with decapsidation of particles of different size could participate in the temporal regulation of gene expression. Unfortunately, this possibility has been thus far proposed for BMV only, and further work will be required to establish whether this is a general feature of multipartite viral systems.

**Fig 2:**
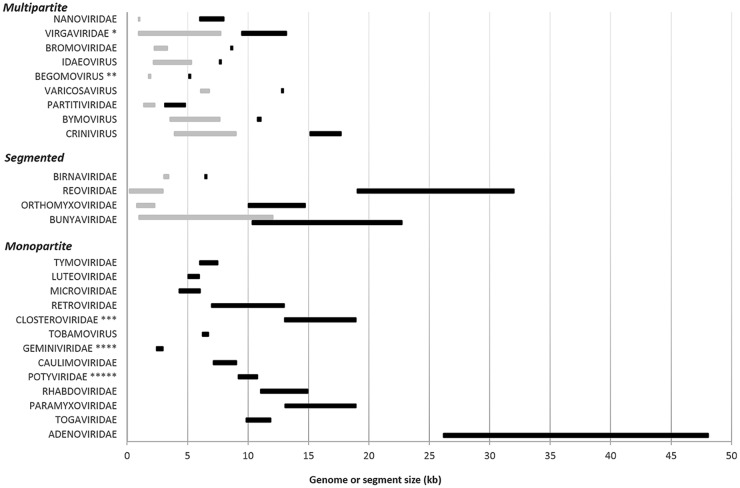
Comparison of the genome or segment sizes in the three types of viral genome organization. The listed families or genera are those with significant differences in genome architectures. Not all viral families are represented in this figure. We have chosen families with a large range of total genome sizes, but avoiding those monopartite virus groups with an immense genome that would have compressed too much the scale of the graphic. We also show viral families containing both mono- and multipartite member species. The size range of whole genomes and that of individual segments are illustrated by black and grey lines, respectively. All size data come from the website (http://viralzone.expasy.org/), from the ninth International Committee on Taxonomy of Viruses (ICTV) report, and from specific literature. *All genera of the family *Virgaviridae* except the genus *Tobamovirus* are composed of multipartite virus species. **The genus *Begomovirus* is composed of both monopartite and bipartite virus species. ***All genera of the family *Closteroviridae* are composed of monopartite virus species, except for the genus *Crinivirus*. ****All genera of the family *Geminiviridae* are composed of monopartite virus species, except for the genus *Begomovirus*. *****All genera of the family *Potyviridae* are composed of monopartite virus species, except for the genus *Bymovirus*.

Perhaps related to similar questions of encapsidation constraints is the report on the experimental evolution of *Foot and mouth disease virus* (FMDV) [14]. Through repeated passages in cell cultures at elevated multiplicity of infection, Ojosnegros and colleagues observed that two defective molecules complementing each other could outcompete their ancestral monopartite FMDV. The selective advantage of this bipartite derivative was demonstrated to be associated with a higher stability and infectivity of the virus particles packaging smaller RNA segments, and not to a faster replication. The authors thus proposed that genome segmentation and, more specifically, multi-encapsidation could in some cases result from a trade-off between segment length and particle stability. While it has been shown that particle size was correlated to genome length [76], there is no clear general correlation between genome length and particle stability. For this reason, we believe that the case of segmented FMDV variant taking over the monopartite parental genome might be a specific example, related to experimental conditions, and hardly expandable as a general rule to explain the evolution of multipartite viruses. Perhaps consistently, it should be noted that this FMDV experiment was conducted with a virus of vertebrates, in which no multipartite natural systems have ever been described. Finally (and this might be a most definitive argument pleading against the genome multi-encapsidation as a way to preserve particle stability for oversized genomes), (i) multipartite viruses do not necessarily have longer genomes than monopartite viruses, and (ii) they sometimes encapsidate segments that are longer than the whole genome of some monopartite viruses (Fig 2).

### 5. Within-host movement

When viruses move from cell to cell or across long distances to systemically colonize their host, the question of transferring all of their genome information becomes a real issue distinguishing multipartite viruses from both segmented and monopartite. Indeed, for the latter two, the whole genetic information may move as a whole, packaged as complete information units within individual virions. In contrast, multipartite viruses package their genetic information in distinct virus particles, which must somehow come together to initiate infection. The question in this section is, thus, to see whether some striking specific features emerge in the known mechanisms of within-host spread for multipartite viruses.

The available molecular and cellular data show that viral trafficking within the host plant is multifarious (reviewed in [70,77]). Some plant virus species move both cell-to-cell and long distance as mature virus particles. Others can move cell-to-cell as nucleoprotein complexes not assembled into mature virus particles, which are only required for long-distance movement. Finally, in rare cases, some viral species can spread both cell-to-cell and in the plant vasculature as nucleoprotein complexes that do not even contain the coat protein. All three cases have been suggested in both monopartite and multipartite viruses [78]. For example, monopartite *Cauliflower mosaic virus* [78] and multipartite *Cowpea mosaic viru*s [79, 80] move both cell-to-cell and long distance as mature virions. On the opposite end, both the monopartite *Tomato bushy stunt virus* [81] and the bipartite *Cabbage leaf curl virus* [82] move cell-to-cell and long distance without the coat protein.

**Fig 3:**
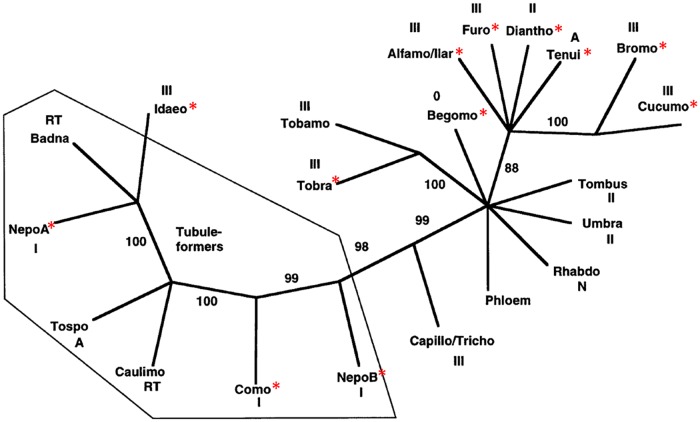
Relationship between the phylogeny of movement proteins of the 30K superfamily and the genome organization of corresponding viruses. This tree was constructed from all movement protein sequences available at the time of reference [85] using parsimony analysis. For more details on the construction of the tree, see reference [85]. 0, RT, N, A, I, II, and III represent the type of polymerase encoded by the viruses: none, RNA-dependent DNA polymerase, negative-strand virus, ambisens-strand virus and positive-strand virus, and supergroups I, II, III RNA-dependent RNA polymerases, respectively. The thin-lined polygon encloses those movement proteins known to form virion-bearing tubules. Genera with a red asterisk are those whose member species are multipartite viruses (N.B.: The genus *Begomovirus* is composed of both monopartite and bipartite viruses). Reproduced and adapted from reference [85].

Whatever the viral form that is actually transported, with regard to limitations linked to the size exclusion limit of plasmodesmata, plant viruses have developed different specific mechanisms to ensure their passage to the adjacent cells and into the vasculature, all depending on one or more movement proteins. Through intricate interactions with multiple host factors, some of these movement proteins enlarge the size of plasmodesmata, allowing the passage of infectious material, whereas others polymerize into a tubular structure acting as a syringe to inject the virus into the neighboring cells [83,84]. These distinct modes of action are shared by multipartite, segmented, and monopartite viruses (reviewed in [77]), and, thus, the present data do not distinguish multipartite virus movement mechanisms from those of other viruses. One further illustrative example of this conclusion is the phylogeny of the viral movement proteins of the 30K super family, established by Melcher in 2000 [85]. In Fig 3, it is apparent that the relatedness between these 30K movement proteins does not depend on the genome organization of the corresponding viruses.

If no distinct molecular or cellular features appear in the mechanisms of within-host movement of multipartite viruses, perhaps they differ in the number of virus particles or genomes that actually enter and infect individual cells (multiplicity of cellular infection [MOI]). For monopartite viruses, the independent action hypothesis (IAH) stipulates that infection of a cell and/or host can be initiated by a single infectious unit, and that each infectious unit can act independently [86] (reviewed in [87]). Theoretical models predict deviations from IAH when one infecting component depends on the presence of others, and indicate that the number of viral particles efficiently entering a cell (here simplified as MOI) must be much higher in multipartite viruses than in monopartite or segmented ones. The MOI allowing for the maintenance of genome integrity is predicted to be directly related to the number of genome segments and to their relative frequencies and should reach very high values (up to hundreds) when multipartite viruses have more than three or four segments [15].

MOI values have been experimentally estimated in a number of monopartite viruses infecting bacteria [88], insects, vertebrates, and plants (reviewed in [87,89]), and repeatedly found to be relatively small, most often below five. Unfortunately, there is only one estimate of the MOI of a multipartite virus, the bipartite *Soil-borne wheat mosaic virus*, in which only RNA-2 segment was analyzed [90]. The estimated value is similar to that in monopartite viruses, in the order of five, not supportive of the prediction that multipartite viruses infect cells with higher numbers of virus particles or genome segments. Due to the paucity of data, and considering the importance of this question in the biology of multipartite viruses, more investigation is needed and should focus on species with a high number of segments, such as, for example, members of the families *Bromoviridae* (3 ssRNA segments + 1 sgRNA) and *Nanoviridae* (6–8 ssDNA segments) or of the genus *Benyvirus* (4–5 ssRNA segments).

### 6. Inter-host movement

The problem of maintaining the integrity of multipartite viral genomes not only questions steps of within-host cell-to-cell or long-distance spread, but also that of host-to-host transmission. In this section, we will focus on horizontal transmission by vectors because this is the major and best-documented mode of transmission of plant viruses. Although data are still lacking in some cases, viruses are generally assumed to be acquired from infected plants and inoculated into healthy ones under the form of virus particles [91]. This extends the risk of scattering and, thus, of losing parts of the genetic information when a multipartite virus is spread in the host population. That vector transmission can induce severe bottlenecks into virus populations has been described in monopartite viruses of animals and plants (reviewed in [87,89]). However, cases in which this bottleneck was actually quantified are rare in plant viruses and not yet available in animal viruses. The transmission of *Potato virus Y* (PVY) was shown to result from the transfer of as few as one or two genomes per aphid vector [92], and similar figures were obtained for the whitefly-transmission of *Tomato yellow leaf curl virus* (TYLCV) [93]. Higher numbers are intuitively expected for multipartite viruses, in order to increase the chances of co-transmitting a set of particles containing at least one copy of each segment. Surprisingly, when quantifying the effective number of founders that initiate an infection after aphid transmission of the tripartite *Cucumber mosaic virus* (CMV), Betancourt and colleagues [94] found numbers similar to those mentioned above for PVY and TYLCV. It should be noted, however, that the methodology used allowed the estimation of the genetic bottleneck but not that of the demographic bottleneck, which is the relevant one for the issue addressed here. In other words, while the transmission-related genetic bottlenecks suffered by viral populations appear comparable in mono- and multipartite viruses, the actual number of viral particles that needs to be acquired and inoculated might be different and remains to be evaluated and compared (discussed previously in [89,94]).

These results open a number of questions, which are thus far unanswered in the transmission of multipartite viruses, and which represent appealing lines of research for the future. In particular, that distinct genome segments accumulate at very different frequencies within host plants [50,56] poses the question of the transmission of the rare segments. Likewise, because virus particles containing distinct genome segments may vary in stability [75], they might well be differentially degraded during the passage within the insect vectors [95], questioning whether the relative frequency of the segments may change within vectors and how the most labile particles can be transmitted as efficiently as the others. Finally, the bottleneck related to vector transmission in the wild could be alleviated by high vector population density and repeated inoculation. Unfortunately, as discussed in the next section, the ecology of multipartite (and other) viruses is poorly known, and this possibility is not sufficiently documented.

### 7. Ecology of multipartite viruses

As far as we are aware, there is no study on specific ecological features that may be associated with multipartite viruses. A large body of literature reports on the geographical variations in genetic diversity of viruses in general and of multipartite viruses in particular. The studies on the frequency of reassortants in the natural population of CMV [45,46], of nanoviruses [37,38],[31], and of bipartite geminiviruses [96] are certainly a strong step in this direction, but they thus far have not revealed any specific ecological traits. Similar to recombinant genomic stretches in other virus types (monopartite or segmented), it clearly appears that different segments of multipartite viruses can have distinct evolutionary histories and distinct phylogeographies [96]. We believe that key information will arise from specific investigation on the circulation of segments of multipartite viruses in the community of host and vector species in a restricted geographical area.

### 8. Concluding remarks and future prospects

The perusal of the literature presented above illustrates our current ignorance on the reasons explaining why multipartite viruses are so successful. The simple fact that they can be ssRNA, dsRNA, or ssDNA likely indicates that multi-encapsidation has evolved more than once, and yet we are unable to confirm any associated beneficial aspects. The proposed putative benefits are not yet convincing because of a lack of data, and because most also appear valid for segmented virus, thus not explaining multi-encapsidation. We believe further experimental work on the specifics of the biology of multipartite viruses is necessary to evaluate and challenge the existing hypotheses, and, even better, propose new ones perhaps more pertinent or unforeseen. A possibility that should not be ignored is that, because multipartitism most likely evolved independently several times, its evolution may have responded to distinct selection pressures: it is possible that the reasons that led to the evolution of multipartitism differ in different groups of viruses and that potential benefits that exist in one group do not exist in another. As concluding remarks, we outline a few research lines that could clarify or assign specific properties to multipartite viruses that we judge immediately critical.

Structural, physical, and biochemical properties of the virus particles depending on the contained segment(s) should be investigated in more detail. An important outreach of these studies (detailed in section 4) is that distinct properties of particles containing different segments may reflect an adaptive process involved in the temporal regulation of gene expression specific to multipartite viral systems. Although such variable particle properties were shown to be related to the RNA folding structure, they may also be important for multipartite ssDNA viruses in which secondary/tertiary folding structures of various segments appear to have unknown biological functions [97].

Also related to the regulation of gene expression, the discovery of the genome formula in populations of the nanovirus FBNSV [50] contributes to the consideration of a putative important role of gene copy number variations in the biology of viruses. Prominent questions are whether the genome formula is also controlled in other multipartite viruses, whether it actually regulates gene and phenotype expression, and whether it is an adaptive and evolvable trait. While arguments in favor of the adaptive regulation of gene copy number in multipartite viruses are discussed in section 3, a direct experimental demonstration is still lacking.

To maintain the integrity of multipartite viral genomes, there are two undecided possibilities. First, particles could massively penetrate cells with whatever probability independent of the identity of the contained segment. Second, multipartite viruses could somehow sort particles that enter a cell depending on the encapsidated segment and promote the selective entry of complete sets of the viral genetic information (interesting mechanistic discussion on this point is found in [70]). In the first scenario, the observed MOI values for the different segments should be related to their frequency within the viral population infecting an individual host (high for frequent segments and low for rare ones). In the second scenario, the MOI values might be low and should be non-correlated to the segment frequencies within the population. This understudied aspect would be very informative on the way of life of the multipartite viruses.

Ultimately, the most important view to be incontrovertibly verified is that all segments of a multipartite virus need to be together in the same host cell for the system to be functional. This dogmatic assumption forms the basis of the cost always attributed to the multipartite life-style. Surprisingly, it has never been experimentally verified. Experimentally assessing whether such viral systems could function with their different segments scattered in different cells is tempting because of recent progress in plant physiology. Many studies are reporting the capacity of specific proteins [98],[99] and RNA [100–102] to traffic autonomously from cell-to-cell or long distance within host plants. Assuming that some RNA and/or proteins of multipartite viruses can traffic on their own opens the possibility for a viral function to efficiently act within cells devoid of the gene encoding it. In this view, the reduced chances to infect individual cells with all segments together would no longer be such an acute problem, and the angle with which the biology of multipartite viruses could best be conceptualized would be noticeably different.
